# Surgeon perception of factors affecting the efficiency of conventional and robotic laparoscopy: A Pan India study

**DOI:** 10.1016/j.heliyon.2022.e12561

**Published:** 2022-12-24

**Authors:** Prabhat Kumar, Radhik Rammohan, Mrunal Chavan, Rupesh Ghyar, Suresh Deshpande, Jagdeesh N. Kulkarni, Hemant Bhansali, Bhallamudi Ravi

**Affiliations:** a Mechanical Engineering Department, Indian Institute of Technology Bombay, Mumbai, India; b Electrical Engineering Department, Indian Institute of Technology Bombay, Mumbai, India; c Biomedical Engineering & Technology Innovation Centre, Indian Institute of Technology Bombay, Mumbai, India; d Swarup Hospital, Kolhapur, India; e Minimal Invasive and Robotic Surgery, Asian Cancer Institute, Cumbala Hill Hospital, Mumbai, India; f Nanavati Hospital, Mumbai, India

**Keywords:** Laparoscopy, Robot-assisted surgery, Minimally invasive surgery

## Abstract

**Background:**

Laparoscopic surgery, being minimally invasive, offers many benefits including faster patient recovery, reduced scarring and lower mortality rate. It is, however, technically challenging and requires a long learning curve. These issues can be overcome by Robot-Assisted Surgery (RAS) systems, which incorporate computer-controlled motions enabling enhanced precision and accuracy.

**Methods:**

This study involves identifying and verifying various difficulties related to laparoscopy and the role of RAS in their mitigation. It involved 93 surgeons across India, covering a range of demographics, medical specialties and experience. They were interviewed to understand the current status and to compare RAS with conventional laparoscopy. The questionnaire developed for the purpose tests a set of hypotheses related to instruments, comfort, and other factors derived from the available literature as well as inputs from leading laparoscopy surgeons and domain experts.

**Results:**

A grading system was adopted to evaluate the hypotheses based on the surgeons' responses. A statistical method based on T-test was employed to gain useful inferences from the study. The results showed that early-career surgeons preferred haptic enabled systems. As the experience of the surgeon increases, tissue identification becomes easier, thereby reducing the need for haptic feedback-enabled instruments.

**Conclusions:**

The surgeons from across the demographics were strongly in the favour of the need for articulated instruments with surgeon-controlled camera systems. They reported a reduction in physical and mental discomfort during surgical procedures using RAS. They also confirmed the similarity in patient outcomes for both conventional laparoscopy and RAS. These insights are expected to be interesting and useful for further research and development in this field.

## 1 Introduction

**Figure 1:**
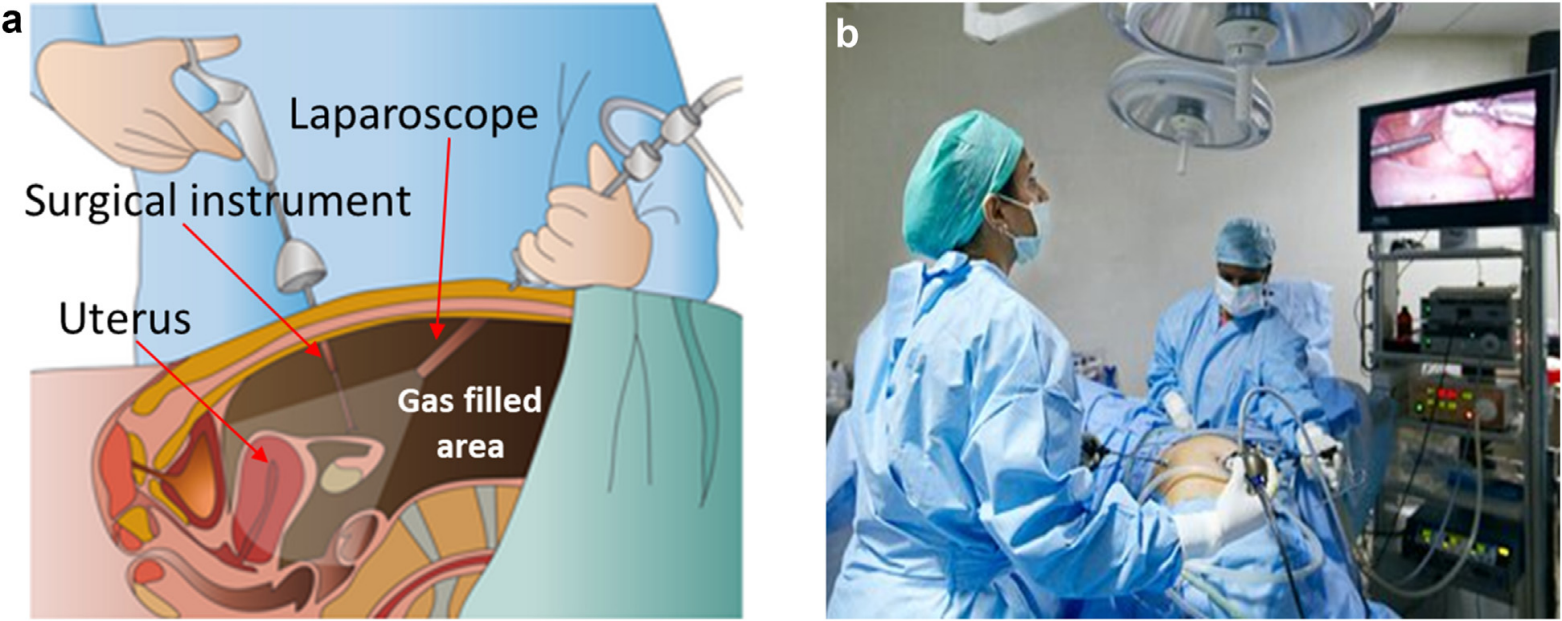
(a): Laparoscopic surgery, (b): Surgeons viewing inside the abdomen on screen [2].

Laparoscopic surgery is a minimally invasive surgery (MIS) technique [1], where small incisions are made in the abdomen of a patient to insert instruments for various surgical tasks. Through one such incision, a camera is also introduced for visual feedback of the area of interest (Figure 1 (a), (b)) [2]. This method of surgery has generally led to reduced scarring, faster patient recovery and shorter hospital stays. However, the method has its own challenges too [3]. Surgeons lose the touch and feel of the tissues, thus increasing cognitional difficulty in tissue characterization. Many of them develop pain in shoulder, neck and wrist as the surgery extends to prolonged duration (more than 3 h); the higher physical effort also affects the speed and efficacy of the surgery.

**Figure 2:**
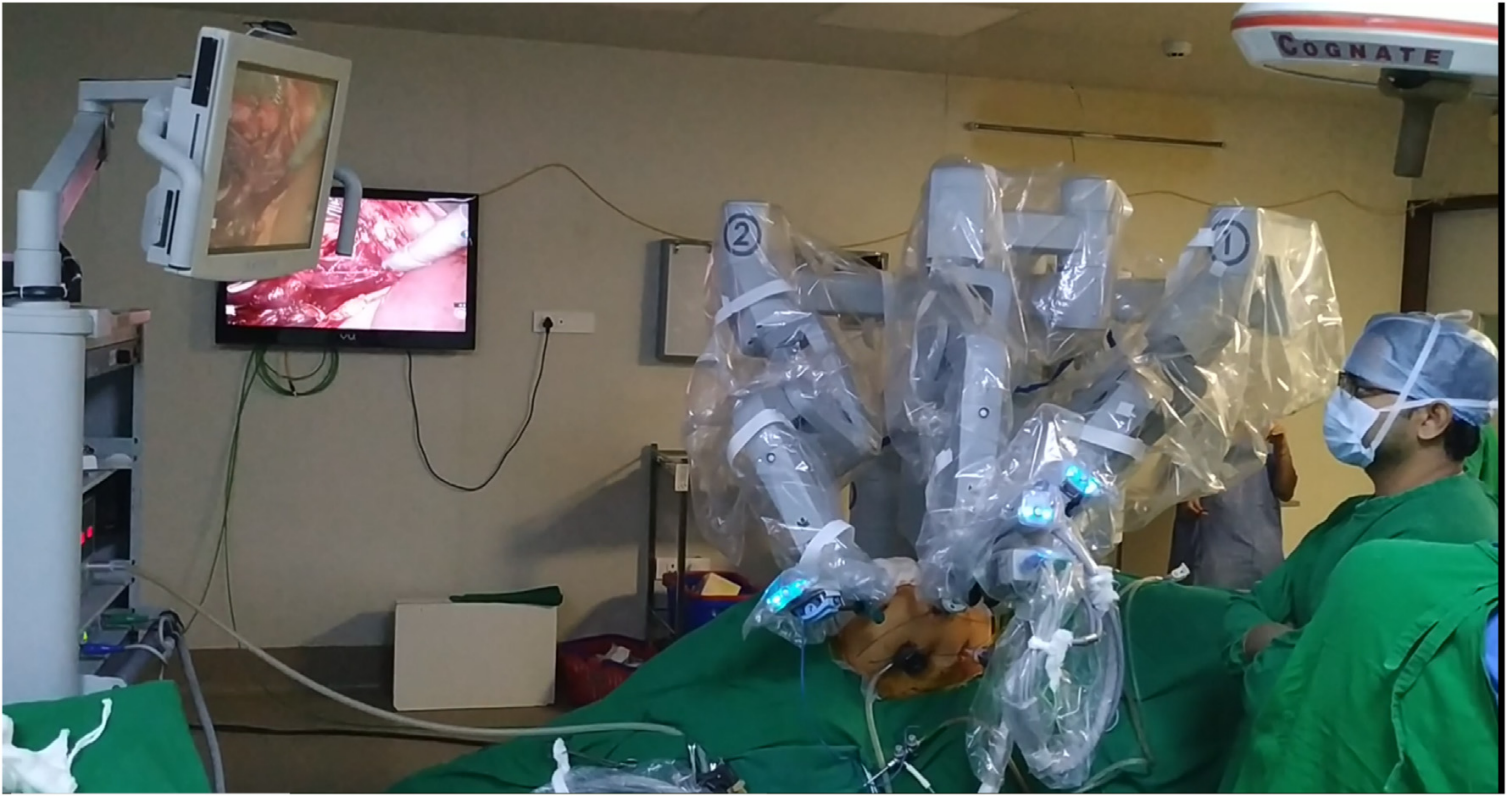
Robotic surgery using Da Vinci system.

Robot-assisted surgery systems were developed to overcome such challenges. Robotic manipulators have made laparoscopic surgeries relatively easy and intuitive compared to conventional methods. Among various mechanisms for surgical robotic manipulators, wire-driven devices have become popular. The most representative example is the EndoWrist used in da Vinci Surgical System (Figure 2) from Intuitive Surgical [4]. Such systems have a shorter learning curve, compared to conventional laparoscopy, along with higher maneuverability and better ergonomics [5, 6]. However, these systems are expensive and require significant changes to the operating room prior to deployment [7].

Since the introduction of RAS in 2000, a number of international researchers reported on differing levels of understanding and acceptance of this technology among surgeons and patients in a number of surgical specialties such as urology and gynecology. Limited data exist regarding the knowledge and perceptions of surgeons about RAS in the developing countries, especially in India. This study aimed to understand the current usage, challenges and unmet needs of laparoscopy surgeons, particularly regarding the characteristics and effectiveness of conventional instruments and robotic-assisted surgery systems. It involved the survey of surgeons who have experienced urological, gynecological and general laparoscopic surgeries to identify their perceptions about the unmet needs in this domain.

### 1.1 Literature review

Robot-assisted surgery systems enhance surgeon capability by improving the positional and motion control of the surgical instruments [8]. The 3D vision system enhances the tissue characterization ability of the surgeons. The physical discomfort encountered by surgeons in conventional laparoscopy is reduced by the robotic systems, which are ergonomically superior. Currently available RAS systems include Da Vinci from Intuitive Surgical (Sunnyvale, CA, USA) [9]; Versius from Cambridge Medical Robotics (CMR) Surgical (Cambridge, United Kingdom); and Hugo from Medtronic (Watford, UK). The RAS systems however, have several disadvantages such as high cost, lack of haptic feedback, higher consumption of operation theater space, and lack of variety of instruments [10].

It has been observed by other researchers that robotic surgery is not necessarily more expensive than open and laparoscopic surgery when all influence factors are taken into account [11]. Even for demanding visceral surgery procedures, the perioperative complication rate for robotic surgery is not higher than that for open or laparoscopic surgical procedures. In cancer cases, the oncological accuracy of robotic resection for gastric, pancreatic and rectal resection is seen to be adequate [1, 11]. While the operating time is generally longer for robotic surgery than for standard laparoscopic and open procedures, robotic surgery is found to result in reduced blood loss, lower conversion rates, and shorter hospital stays in some procedures.

The challenges faced by laparoscopy surgeons have been reported in a few studies. The results of a survey based on 241 urology surgeons in China show that the surgeons were suffering from varying degrees of physical discomfort caused by ergonomic issues [12]. Another study conducted in Bangalore, India assessed the response of 150 laparoscopy surgeons using a questionnaire on demographic information, ergonomic issues in the operating room, musculoskeletal symptoms, etc. [13]. The respondents experienced discomfort in the following regions in descending order: right shoulder (87.2%), left shoulder (78.3%), neck (67.4%), back (46.8%), hand (30.3%), leg (21.6%), and wrist (15.8%). The respondents with more than five years of experience complained less discomfort than those with less than five years. Only 54.6% of respondents were aware of the relevant ergonomic guidelines.

Ergonomic assessment of laparoscopy, laparo-endoscopic single-site surgery (LESS), and robot-assisted surgery showed that the surgeon’s posture during laparoscopic surgery is mainly affected by static body posture, height of the operating table, design of the surgical instruments, position of the main screen, and the use of foot pedals [14]. A study conducted with 68 members of Canadian Hepatopancreatobiliary Association (CHPBA) showed that most of the HPB surgeons use laparoscopy for minor HPB resections [15]. While there is a strong desire to expand the use of minimally invasive techniques, training is needed to address the limitations and barriers to increased use of minimally invasive procedures. Also, Pickett et. al performed a study by conducting a survey to compare opinions of general and subspecialty obstetricians and gynecologists regarding teaching robotic surgery (RS) to residents [16]. One similar study was conducted on 38 patients in an ambulatory general surgery clinic in which it was highlighted that novelty had an important influence on patient preference for robot-assisted surgery [17].

Another survey was conducted on Kuwait’s surgeons and patients aimed to explore their perceptions about RAS [18]. It involved distributing a questionnaire among surgeons of different specialties as well as patients. Their self-reported experience with technology was related to the level of comfort with computers and computer literacy. Most surgeons had heard about RAS, and the majority agreed to its introduction into the healthcare system. However, 27% of them believed that the surgeon has complete control over the robot, and 25% were uncertain about the level of control, which reflected poor knowledge about this technology. This study showed that the surgeons and patients had limited knowledge and perceptions about RAS, owing to the absence of visual and social media platforms compared to their Western counterparts. Most of the studies mentioned above were carried out using questionnaire-based surveys. It has been reported that this method leads to response bias, especially when the responses include leading answers which don’t reveal the actual opinions of respondents [19].

One study was conducted at a robotic surgery performing hospital in New York, USA where the authors administered survey questionnaires via face-to-face interviews with patients, healthcare providers and senior members of hospital administrations [20]. It was found that there is a discrepancy between the perceptions of robotic surgery and the clinical evidence among the respondents of the survey. This survey was conducted in only one hospital, limiting the validity of the results over a diversified population.

The above studies have been carried out in various developed countries across the world, including USA, Canada, Europe and China. There have been very few studies in developing countries. In particular, India is witnessing a rapid growth in both conventional and robot-assisted surgeries. This makes it necessary to understand the perceptions and experiences of Indian surgeons in using RAS and the present study involving a survey of 93 laparoscopy surgeons from various parts of India aims for the same.

## 2 Materials and methods

In order to understand the current usage, challenges and unmet needs of laparoscopy surgeons, the authors visited and interacted with 93 laparoscopy surgeons from the different parts of India covering many places from North to South, East to West and Central India too. The surgeons represented a range of specialties (general surgery, gynecology, urology, pediatric, etc.) and experience (novice to expert level).

The study provides the perceptions of the surgeons about the laparoscopic surgical procedures which was graded by the authors who had significant statistical expertise. Thus, this study falls in the category of minimal to no risk and hence Institutional Review Board (IRB) approvals were not taken. However, an informed verbal consent was taken from all the participants prior to the interviews. The participation of the surgeons in the survey was completely voluntary.

A set of sub-hypotheses, which revolved around a central hypothesis, (described in a later section) about laparoscopic surgery and instrument characteristics were prepared based on existing literature [21], followed by the formulation of a set of questions. The surgeons were interviewed in person and their answers were graded in ascending manner, with 1 point allotted for ‘strongly disagree’ to 5 points for ‘strongly agree’. The interview data was compiled in appropriate formats to statistically analyze the results. These were utilized to derive the list of desired features of robot-assisted surgical system. The above steps are described in detail here.

### 2.1 Survey demography

To ensure a wider representation, various laparoscopic surgeries performed in India were reviewed and the top five were selected based on inputs from multiple stakeholders including hospitals and training centers [19, 20]. Additionally, geographic and ethnic diversity was ensured by involving surgeons from different parts of the country.

#### 2.1.1 Medical specialty of surgeons

**Figure 3:**
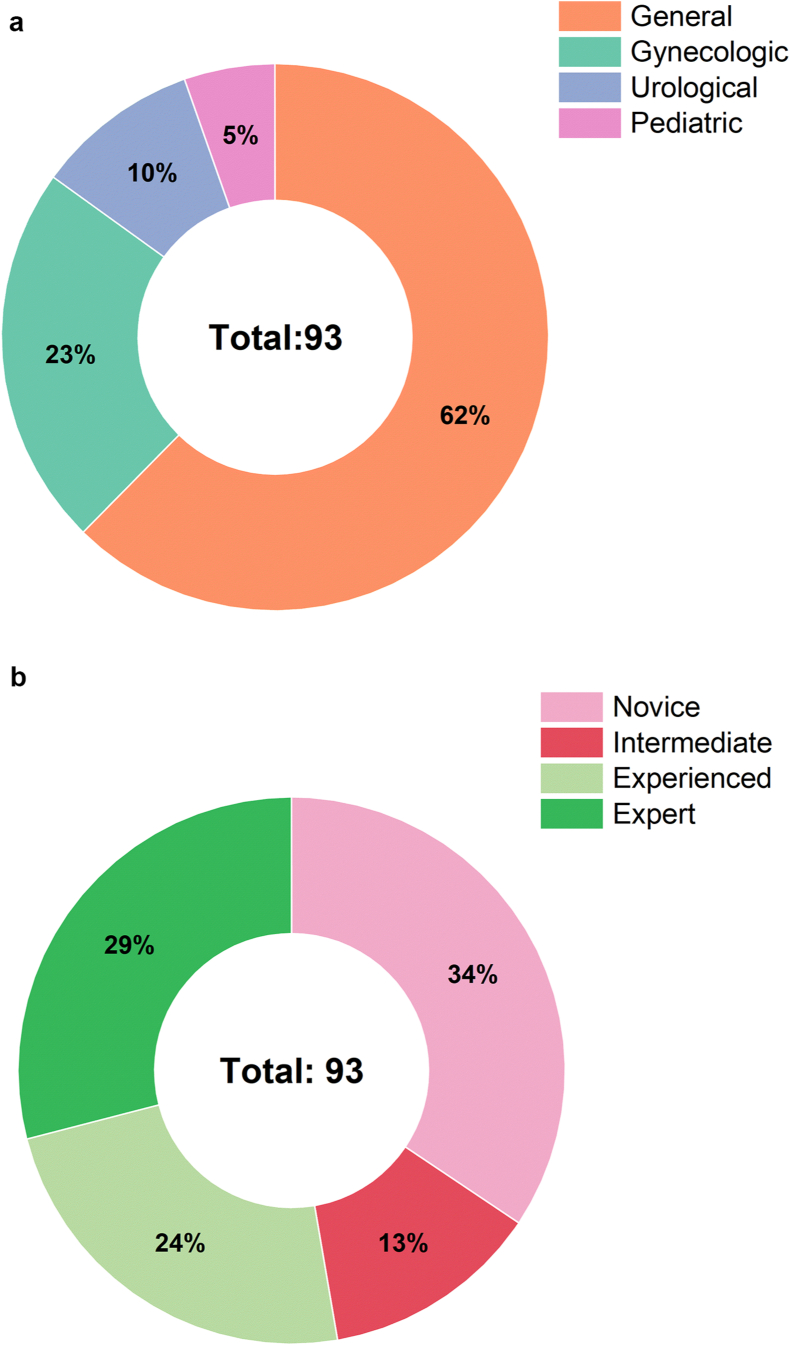
(a): Medical specialty of interviewed surgeons, (b) Expertise level of the interviewed surgeons.

More than 150 surgeons across India were identified and contacted over mail and phone calls to schedule appointments for face-to-face interviews. Out of these, 93 surgeons responded, who were visited in person and interviewed. All interviews were carried out during May–June 2019. The medical specialties of the surgeons interviewed included the following (the percentage of surgeons is indicated in Figure 3(a)).

• **General:** Pancreatic cancer, gallbladder cancer, liver tumors, gastric bypass, bariatric surgery, hernia, gastrointestinal/rectal conditions.
• **Gynecologic:** Benign and malignant tumors, endometriosis, uterine fibroids, ovarian cysts, cervical disorders, hysterectomy, removal of ovaries, and lymph node staging.
• **Urological**: Kidney disorders, kidney cysts, kidney stones, kidney blockage, kidney donation, incontinence, prostate cancer, and vaginal prolapse.
• **Pediatric:** Surgeries involving fetuses, infants, children, adolescents and young adults.

#### 2.1.2 Level of expertise

The laparoscopy surgeons interviewed in this study represented both conventional laparoscopy and robot-assisted procedures, with the non-robotic group in the majority (70%). The conventional laparoscopy surgeons interviewed in the survey have either gone through the training of robotic surgeries or assisted surgeons during the robotic surgeries. This justifies their opinion about robotic systems, though they are currently working entirely using conventional laparoscopy devices. The expertise level of the surgeons is represented by the number of laparoscopic procedures performed annually, which is adapted from the existing literatures [21, 22]. The percentage of surgeons interviewed in each level is indicated in Figure 3(b).

• **Novice**: Surgeons who do not have any prior experience in laparoscopic surgery.
• **Intermediate**: Surgeons performing 10–100 laparoscopic procedures.
• **Experienced**: Surgeons performing 100–200 laparoscopic procedures.
• **Expert**: Surgeons who perform more than 200 laparoscopic procedures.

Data about the types of hospitals was also collected. The majority of the surgeons worked in private hospitals (67); nine surgeons were from government hospitals, and 17 were from laparoscopic surgery training centers. Among those in private hospitals, there were 33 surgeons from multi-specialty hospitals and 17 were from super-specialty and general hospitals each.

### 2.2 Hypothesis formulation

**Figure 4:**
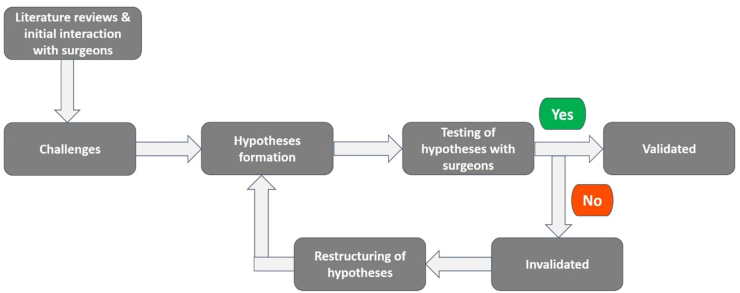
Flow diagram of the methodology.

The study explores the unmet clinical needs of conventional as well as robotic laparoscopy surgeons. For this purpose, a central hypothesis was identified first, backed by five different sub-hypotheses (to be either validated or invalidated). The overall methodology involved formulation of the hypotheses, followed by their testing, restructuring (if needed), and validation, shown in Figure 4.

Based on the review of related research articles and initial interactions with laparoscopy surgeons, a questionnaire was developed to collect the relevant data [21]. The questionnaire aimed at assessing the perceptions of the surgeons about various parameters related to conventional laparoscopic surgery and RAS. Questionnaire items included requirements of articulated instruments, need of assistant for holding camera, haptic feedback in instruments, ergonomics of surgeons during surgery and patient outcome. The questionnaire was prepared regarding both conventional laparoscopic surgeries and RAS techniques. The central hypothesis was identified around which five sub-hypotheses were identified, and further verified by domain experts.

#### 2.2.1 Central hypothesis

The efficiency of the laparoscopic surgeries is affected by various factors such as articulated instruments, surgeon-controlled camera and haptic feedback.

This hypothesis is categorized into five different sub-hypotheses which were tested for validation/invalidation. These five sub-hypothesis are explained below in detail.

#### 2.2.2 Articulated instruments hypothesis

Articulated instruments are needed for better reach, accessibility and maneuverability for the area of interest. The MIS instruments have only five degrees of freedom, but a complicated procedure like suturing requires seven degrees of freedom [23, 24]. Surgeons require more flexibility of the devices, especially when time is of the essence. To reach the entire operation workspace, laparoscopy surgeons need instruments with more degrees of freedom, thus providing better reach, accessibility, and maneuverability.

#### 2.2.3 Camera hypothesis

Surgeon controlled optical camera is required to eliminate uncertainties associated with surgery assistants. While operating on patients, laparoscopy surgeons have to continuously instruct their assistants to adjust the camera for a proper view of the site of interest. This coordination plays a vital role during complicated operations and exacerbates the mental stress of the surgeons. Hence the surgeons require cameras that can be directly controlled by them to eliminate the dependence on the assistants.

#### 2.2.4 Haptic feedback hypothesis

Haptic feedback is needed to obtain a realistic feel of tissues during the surgery. With conventional laparoscopic surgery instruments, the surgeons lose the sense of touch and are left with only visual feedback, leading to increased mental stress. The haptic feedback helps surgeons to use their past experience for distinguishing between malignant and benign tissues. Thus, incorporating haptic feedback in laparoscopy instruments can provide considerable advantage to the surgeons.

#### 2.2.5 Comfort hypothesis

A high level of mental and physical comfort is provided by robotic surgery. Many surgeries continue for hours at a stretch. During this time, the surgeons may have to use the laparoscopy instruments in physically uncomfortable positions, eventually leading to problems like tennis elbow and cervical spondylitis [13]. The physical discomfort can affect the mental acuity of the surgeons, which has to be maintained at a high level while performing complex surgeries. New advancements in operation techniques, including robot-assisted surgeries, need to consider the physical and psychological comfort of the surgeons.

#### 2.2.6 Patient outcome hypothesis

The patient outcome is better in robotic surgery as compared to conventional laparoscopic surgery. Patient outcome of laparoscopic surgery can be measured in terms of the scar size, blood loss, conversion rate to open surgery, speed of recovery, number of days of hospital stay, etc. These are crucial factors in comparing robotic surgery with conventional laparoscopic surgery.

### 2.3 Initial validation of hypothesis

Once the hypotheses were finalized, the authors conducted an initial validation of these hypotheses. This validation was performed by having discussions with the highly experienced laparoscopy surgeons (total = 10). As per their suggestions, one hypothesis was invalidated, which is the patient outcome hypothesis.

According to the surgeons, the patient outcome does not vary between robotic and conventional robotic surgeries. In both the cases, the surgeons make sure that the patient will get the same results in terms of recovery, blood loss, scar size, etc. Thus, the patient outcome hypothesis was changed and restructured as shown in Table 1. After its restructuring, all five hypotheses were evaluated based on a statistical method which is explained in detail in the next section.

**Table 1:** Hypothesis restructuring based on validation with surgeons.

Sr. No.	Initial hypothesis	Restructured hypothesis
1.	The patient outcome is better in robotic surgery as compared to conventional laparoscopic surgery.	The patient outcome is the same in robotic and conventional laparoscopic surgeries.

### 2.4 Evaluation methodology

Once the hypotheses were finalized and data were collected, the next step was to analyse the data. At first, the recorded data was quantified based on the scoring described below.

#### 2.4.1 Scoring of data from surgeons

The hypotheses were quantified by recording the surgeons' responses as a rating (on 1–5 scale) as illustrated in Table 2. The complete data was quantified on the basis of the rating for each hypothesis (given in Appendix 1 and 2).

**Table 2:** Different scoring criteria with examples.

Score 5	
**Criteria**	The surgeon strongly agrees with the hypothesis and, even provides evidence by stating various case examples to emphasize the opinion.
**Example**	*Surgeon*’*s response for* ‘*Comfort*’ *hypothesis,* As I do a large number of surgeries, which generally take about 2 h on an average, I experience severe physical pain in my elbow and neck region. This mandates me to go for a rest period when I could have done more surgeries. Due to this fatigue, I had once undertaken treatment for Tennis Elbow.
**Score 4**	
**Criteria**	The surgeon agrees with the hypothesis, but believes that it could cease to exist in a few instances.
**Example**	*Surgeon*’*s response for* ‘*Comfort*’ *hypothesis,* *T*he current set of instruments are good to meet our needs, but nothing is enough. The instruments with more capabilities, to reach confined spaces and ability to change space based on organ shape, are welcome.
**Score 3**	
**Criteria**	The surgeon is unable to either confirm or deny the hypothesis. The surgeon’s opinion is that the existence or absence of the hypothesis is equally likely.
**Example**	*Surgeon*’*s response for* ‘*Patient outcome*’ *hypothesis,* While robotic surgery is surely meant to improve the patient outcome, the same has not been evidenced by any literature. Many complicated surgeries can be performed by advanced laparoscopic techniques which are quite a norm and are economic too. So, I can neither deny nor accept the hypothesis.
**Score 2**	
**Criteria**	The surgeon disagrees with the hypothesis but believes that it could potentially exist in some instances.
**Example**	*Surgeon*’*s response for* ‘*Surgeon controlled camera*’ *hypothesis,* There is no specific need for a surgeon controlled camera setup. In our team, we have experienced assistants and they perfectly coordinate with our commands. This setup might be needed in places where there is shortage of experienced hands.
**Score 1**	
**Criteria**	The surgeon completely disagrees with the hypothesis and emphasizes the opinion using examples to prove the point.
**Example**	*Surgeon*’*s response for* ‘*Haptic feedback*’ *hypothesis,* There is no need for haptic feedback. Close your eyes and hold your pen against the table. You will be able to feel the stiff table. Now put your pen on some soft object with your eyes closed. You will definitely be able to feel the softness and texture. Imagine now you have a good quality 3D vision for the same. You actually don’t need the haptic feedback at all.

#### 2.4.2 Statistical evaluation of hypothesis

As the data was collected and scoring was performed, the next step was to evaluate the hypotheses based on a statistical test. For the evaluation of the hypotheses, a statistical test was chosen called a two-sample t-test. It is one of the most commonly used hypothesis tests used to test the difference (d0) between two population means. A typical application is to determine whether the means are equal. In the t-test, the population is assumed to have a normal distribution. Once the samples are believed to have a normal distribution, there is a significance level (α) which determines the level of accuracy of the test. In this study, α = 0.05, which indicates a 5% risk of concluding that a difference exists when there is no actual difference.

**P-value:** The *p-value* is the probability that a data would be at least this much inconsistent with the hypothesis, assuming the hypothesis is true. A low *p-value* means that the data is highly consistent with the hypothesis.

**Figure 5:**
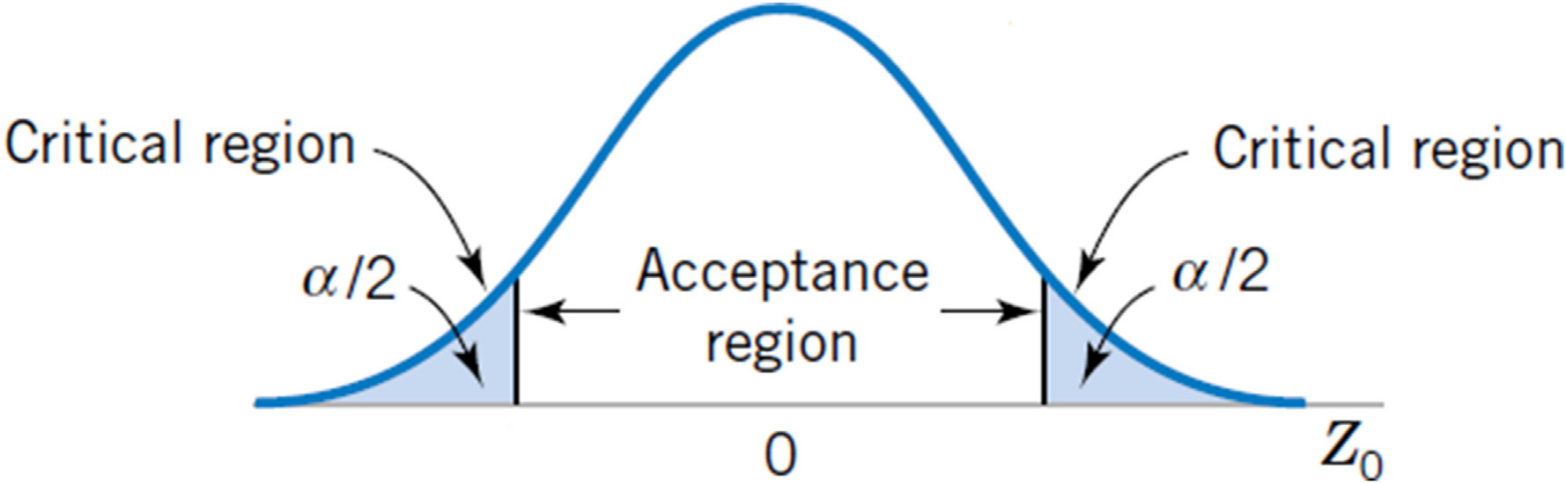
The distribution of Z_0_ when H_0_: μ = μ_0_ is true, with critical region for the two-sided alternative [25].

When the statistical data lies in the acceptance region supported by a *p-value* less than α, the null hypothesis can be accepted (Figure 5). On the other hand, if the result lies outside the critical region (*p value* < α), then it is concluded that the null hypothesis is rejected, and hence the alternate hypothesis is accepted.

In this study, a relevant analysis tool (Two-sample assuming unequal variances) available in Microsoft Excel was employed to calculate the *t-value* and *p-value* (two-tail) for each hypothesis. The underlying assumption in the t-test is that the two populations have normal distributions [26]. For this purpose, the data was divided into two samples: one containing 44 surgeons of less experience in laparoscopic procedures, and the other containing 49 surgeons with a high level of expertise in laparoscopic procedures.

The procedure for the detailed step-wise evaluation is illustrated here for the hypothesis related to the need of articulated instruments.

**Step 1:** Stating the hypothesis
  Hypothesis: Articulated instruments are needed for better reach, accessibility and maneuverability for the area of interest.
  Null hypothesis (H0): The mean difference (for the need for articulated instruments) is zero for both the samples.
  Alternate hypothesis (H1): The mean difference is not zero for both the samples.
**Step 2:** Selecting the significance level.
  An alpha value of 0.05 is considered significant.
**Step 3:** Determining the test statistics.
  Tested against a population hypothesized mean difference (HMD) of 0.
**Step 4:** Comparing the values obtained and interpreting the result.

## 3 Results

The methodology of quantitative evaluation based on the t-test was applied to all hypotheses and the results are given in Table 3. The *t-value* for the need of articulated instruments hypothesis is −2.39, which is lower than the t-critical value of −2.0 (*p-value* of 0.02 which is smaller than the significance level of 0.05). Hence the null hypothesis is rejected with enough evidence, with the hypothesized population mean difference of 0. Thus, we can infer that the need for articulated instruments is not the same for the two groups of surgeons (based on their experience). Also, from Table 3, it can be seen that the mean (4.89 and 4.47) is higher than 4, which implies that both populations endorsed the need for highly articulated devices, but there is a difference in the mean based on the degree of surgeon experience.

**Table 3:** t-test results for different hypotheses.

Hypothesis	Surgeon Experience	Observations	Mean	Variance	HMD	t-stat	t-critical two-tail	P(T ≤t) two-tail
**Articulated instruments**	Less	44	4.89	0.10	0	-2.39	-2.0	0.02
	More	49	4.47	1.31				
**Surgeon controlled camera**	Less	44	4.50	0.67	0	-0.75	-1.99	0.46
	More	49	4.35	1.25				
**Haptic feedback**	Less	44	4.36	0.74	0	-3.87	-1.99	0.0002
	More	49	3.51	1.5				
**Surgeon comfort**	Less	44	4.45	0.39	0	-0.16	-1.99	0.88
	More	49	4.43	0.86				
**Patient outcome**	Less	44	2.86	1.25	0	-1.54	-1.99	0.013
	More	49	2.47	1.99				

For the hypothesis related to the need of surgeon controlled camera, it is found that the t-stat (−0.75) is more than the t-critical (−1.99) with a *p-value* of 0.46 (Table 3), which implies that there is not enough evidence to reject the hypothesis. Hence it can be concluded that there is a need for surgeon-controlled camera for more experienced as well as less experienced surgeons. In the third hypothesis, the need for haptic feedback enabled instruments to understand the stiffness of tissues during surgery was found to be different depending on the experience of the laparoscopy surgeon (p = 0.0002). The *p-value* of 0.0002 implies that there is a sufficient evidence to suggest that the need for haptic feedback is higher for surgeons with higher experience. With increased experience of tissue identification, the need for haptic feedback enabled instruments decreases.

Irrespective of their experience, surgeons agree that haptic feedback enabled instruments are always a ‘useful to have’ feature. For the hypothesis related to comfort, the *t-value* is −0.16, which lies in the acceptance region (tstat < tcritical). This indicates that the surgeon’s mental and physical comfort is more in robotic surgeries (p = 0.88), irrespective of the experience of the laparoscopy surgeon, on the average. Finally, the outcomes of robotic surgery are compared to those in conventional laparoscopic surgery (p = 0.013) for the last hypothesis. Since the population average is less than 3 with a *p-value* less than the significance level (0.05), it can be concluded that both robotic as well as conventional laparoscopic surgeries give similar patient outcomes.

To summarize the above study involving five sub-hypotheses, four were validated with significant evidence based on the responses of the surgeons. One sub-hypothesis was invalidated (surgeon comfort) due to the lack of consensus between the two categories of surgeons. This could be attributed to the large number of operations performed by experienced surgeons using conventional methods, and therefore becoming more familiar and accustomed to that alone.

## 4 Conclusions

This study enabled obtaining the views of 93 laparoscopy surgeons in India, representing the most common medical specialties and years of experience, regarding the challenges and future needs of conventional laparoscopy and robot-assisted surgery (RAS). The set of hypotheses formulated for the study covered most of the key aspects, which were related to articulated instruments, surgeon controlled camera, haptic feedback, surgeon comfort and patient outcome.

**Figure 6:**
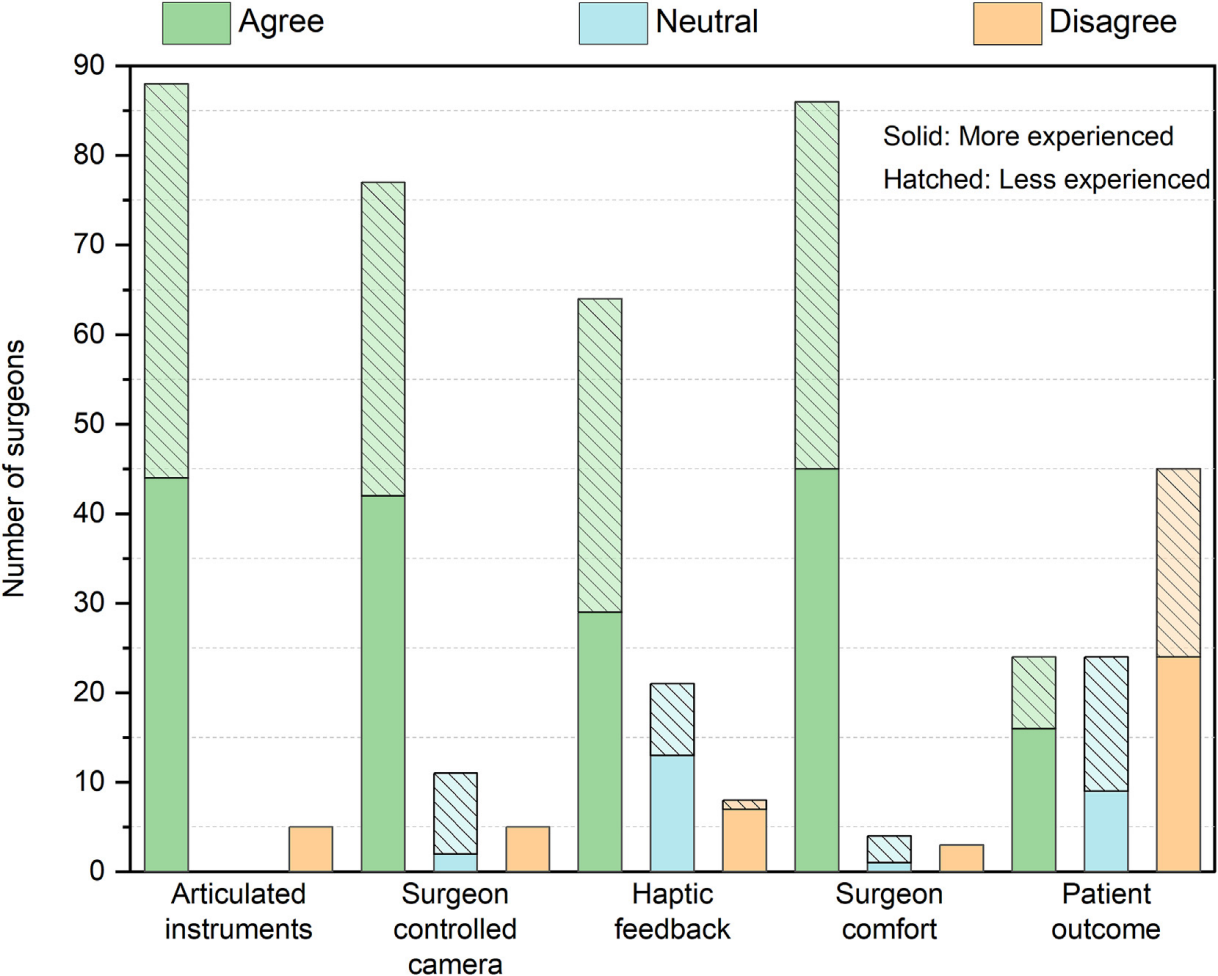
Surgeons' responses on different hypothesis.

According to the surgeons' responses, it was observed that both novice and highly experienced surgeons preferred instruments that allowed complete control over camera movement and a higher degree of articulation (shown in Figure 6). They verified that robotic systems reduced the physical and mental pain associated with conventional laparoscopic procedures. The less experienced surgeons expressed that the design of the conventional laparoscopic instruments needed to be improved. For patient outcome, there is not enough data to prove that robotic surgery is superior to conventional laparoscopic surgery (shown in Figure 6).

To our knowledge, this study is the first to cover a diverse domain and expertise of surgeons from India and attempted to gain their insights by interviewing them in a real-time setting (face-to-face). Strengths of the study include a large-scale survey providing direct feedback from the surgeons performing robotic surgery and getting the perceptions of conventional laparoscopy surgeons. However, the study has some limitations too. In India, the number of hospitals with the robotic surgical system is limited to a few multispecialty centres in major cities [27]. Also, robotic systems are used mainly in advanced laparoscopic procedures. Hence, a majority of surgeons are not exposed to robotic surgery, especially those working in rural setting. Thus, a considerable difference was observed in the opinions of surgeons who work with robotic systems and who only have a perception regarding the same. This study revealed the nature of the demand for robotic systems in India along with the desired benefits. The analysis of the survey is carried out assuming 95% confidence level, and 5% margin of error. We believe our sample (93) is significant and represented the surgical specialties well.

The inferences derived from this study are expected to be useful in defining the user requirements of RAS systems being developed by various research groups. The user requirements can be mapped to functional requirements and design constraints for the instruments and systems, which can provide better outcomes for the patients coupled with higher comfort for the surgeons.

## Declarations

### Author contribution statement

Prabhat Kumar: Conceived and designed the experiments; Performed the experiments; Analyzed and interpreted the data; Contributed reagents, materials, analysis tools or data; Wrote the paper.

Radhik Rammohan: Conceived and designed the experiments; Performed the experiments; Wrote the paper.

Mrunal Chavan: Conceived and designed the experiments; Analyzed and interpreted the data; Contributed reagents, materials, analysis tools or data.

Rupesh Ghyar: Conceived and designed the experiments; Contributed reagents, materials, analysis tools or data.

Hemant Bhansali; Suresh Deshpande Jagdeesh N. Kulkarni: Contributed reagents, materials, analysis tools or data.

Bhallamudi Ravi: Conceived and designed the experiments; Contributed reagents, materials, analysis tools or data; Wrote the paper.

### Funding statement

This research did not receive any specific grant from funding agencies in the public, commercial, or not-for-profit sectors.

### Data availability statement

The data that has been used is confidential.

### Declaration of interest’s statement

The authors declare no competing interests.

### Additional information

No additional information is available for this paper.

## Figures and Tables

**Table:** 

Surgeon	H1:Articulated instruments	H2: Surgeon controlled camera	H3: Haptic feedback	H4: Surgeon comfort	H5: Patient outcome
1	5	5	4	5	5
2	5	5	5	5	3
3	5	5	5	4	3
4	5	5	5	4	3
5	5	5	5	4	3
6	5	5	5	5	5
7	4	5	4	4	5
8	4	5	4	5	5
9	4	5	4	5	5
10	5	5	5	4	2
11	5	5	5	4	2
12	5	5	5	4	2
13	5	5	5	4	2
14	5	5	5	4	2
15	5	5	5	4	2
16	5	5	5	5	2
17	5	5	5	5	2
18	5	5	5	5	2
19	5	5	5	5	2
20	5	4	5	4	3
21	5	5	5	5	2
22	5	5	5	5	2
23	5	4	4	5	3
24	5	5	5	5	2
25	5	5	5	5	2
26	5	5	5	5	2
27	5	3	5	4	3
28	5	5	4	3	3
29	5	5	5	5	2
30	5	5	5	5	2
31	5	5	5	5	2
32	5	5	5	5	2
33	5	5	4	5	5
34	4	4	4	5	5
35	5	5	4	5	4
36	5	3	3	4	3
37	5	3	3	4	3
38	5	3	3	4	3
39	5	3	3	4	3
40	5	3	3	4	3
41	5	3	3	3	2
42	4	3	2	3	3
43	5	3	3	4	3
44	5	4	3	5	2

**Table:** 

Surgeon	H1:Articulated instruments	H2: Surgeon controlled camera	H3: Haptic feedback	H4: Surgeon comfort	H5: Patient outcome
1	5	4	4	5	1
2	4	5	4	4	1
3	4	5	4	4	1
4	5	5	5	5	1
5	5	5	5	5	1
6	4	5	4	5	1
7	5	5	5	5	1
8	5	5	4	4	1
9	5	5	4	5	1
10	5	4	4	4	3
11	5	5	4	5	4
12	5	3	3	4	3
13	5	5	3	4	3
14	5	5	5	5	4
15	1	1	1	1	1
16	5	5	5	5	3
17	4	5	4	5	3
18	4	4	5	4	4
19	5	5	3	4	3
20	1	2	1	2	2
21	5	5	4	4	4
22	5	2	1	3	2
23	5	4	3	5	4
24	5	5	5	5	2
25	5	5	4	5	2
26	1	1	4	4	1
27	1	1	4	4	1
28	4	5	3	5	1
29	5	5	4	5	1
30	5	5	4	5	1
31	5	5	3	5	1
32	5	5	4	5	4
33	5	4	3	1	4
34	5	5	3	5	3
35	5	5	1	4	3
36	4	5	3	5	5
37	5	4	1	5	4
38	5	5	1	5	1
39	5	5	5	5	4
40	5	5	3	5	4
41	5	4	1	4	1
42	5	5	5	5	4
43	5	4	3	5	4
44	5	4	4	4	1
45	2	3	4	5	1
46	5	5	4	5	4
47	5	5	5	5	5
48	5	4	3	5	4
49	5	5	3	4	3
